# Association between hemoglobin and chronic kidney disease progression: a secondary analysis of a prospective cohort study in Japanese patients

**DOI:** 10.1186/s12882-022-02920-6

**Published:** 2022-08-23

**Authors:** Wushan Pan, Yong Han, Haofei Hu, Yongcheng He

**Affiliations:** 1 Department of Nephrology, Kaifeng Central Hospital, Kaifeng, 475000 Henan Province China; 2 Department of Emergency, Shenzhen Second People’s Hospital, Shenzhen, 518000 Guangdong Province China; 3 Department of Emergency, The First Affiliated Hospital of Shenzhen University, Shenzhen, 518000 Guangdong Province China; 4 Department of Nephrology, Shenzhen Second People’s Hospital, Futian District, No.3002 Sungang Road, Shenzhen, 518000 Guangdong Province China; 5 Department of Nephrology, The First Affiliated Hospital of Shenzhen University, Shenzhen, 518000 Guangdong Province China; 6 Department of Nephrology, Shenzhen Hengsheng Hospital, Baoan District, No. 20 Yintian Road, Shenzhen, 518000 Guangdong Province China

**Keywords:** Hemoglobin, Chronic kidney disease, Progression, Non-linear, Cox proportional-hazards model, linear regression model

## Abstract

**Objective:**

Anemia has been reported as a risk factor for chronic kidney disease (CKD) progression. However, there are still few studies examining the relationship between specific hemoglobin (Hb) levels and renal prognosis and renal function decline simultaneously. Meanwhile, the possible non-linear relationship between Hb and CKD progression also deserves further exploration. On that account, our primary goal is to explore the link of Hb on renal prognosis and renal function decline in patients with CKD.

**Methods:**

This study was a secondary analysis of a prospective cohort study, which consecutively and non-selectively collected 962 participants from the research of CKD-ROUTE in Japan from November 2010 to December 2011. We used the Cox proportional-hazards and linear regression models to evaluate the independent association between baseline Hb and renal prognosis (renal composite endpoint, initiation of dialysis during follow-up or 50% decline in eGFR from baseline) and renal function decline(annual eGFR decline), respectively. A multivariate Cox proportional hazards regression analysis with cubic spline functions model and smooth curve fitting (penalized spline method) were conducted to address Hb and CKD prognosis's non-linearity. At the same time, a generalized additive model (GAM) and smooth curve fitting (penalized spline method) was conducted to explore the exact shape of the curve between Hb and renal function decline. Additionally, we did a series of sensitivity analyses to ensure the robustness of the results. Moreover, we conducted subgroup analyses.

**Results:**

The mean age of the included patients was 67.35 ± 13.56 years old, and 69.65% were male. The mean baseline Hb and estimated glomerular filtration rate (eGFR) was 12.06 ± 2.21 g/dL and 33.04 ± 18.01 ml/min per 1.73 m^2^. The annual decline in eGFR was 2.09 mL/min/1.73 m^2^/year. During a median follow-up time of 33.5 months, 252(26.2%) people experienced renal composite endpoint. After adjusting covariates, the results showed that Hb was negatively associated with renal composite endpoint (HR = 0.836, 95%CI: 0.770, 0.907) and renal function decline (β = -0.436, 95%CI: -0.778, -0.093). There was also a non-linear relationship between Hb and renal composite endpoint, and the inflection point of Hb was 8.6 g/dL. The effect sizes(HR) on the left and right sides of the inflection point were 1.257 (0.841, 1.878) and 0.789 (0.715, 0.870), respectively. And the sensitive analysis demonstrated the robustness of the results. Subgroup analysis showed that Hb was more strongly associated with the renal composite endpoint in non-hypertensive, SBP < 140 mmHg, urine protein-to-creatinine ratio (UPCR) < 0.5 g/gCr, and diuretic use patients. In contrast, the weaker association was probed in hypertensive and non-diuretic use patients and the patients with SBP ≥ 140 mmHg, and UPCR ≥ 0.5 g/gCr.

**Conclusion:**

This study demonstrates a negative and non-linear relationship between Hb and renal prognosis and renal function decline in Japanese CKD patients. Hb is strongly related to renal prognosis when Hb is above 8.6 g/dL.

**Supplementary Information:**

The online version contains supplementary material available at 10.1186/s12882-022-02920-6.

## Background

Chronic kidney disease (CKD), which causes end-stage renal disease (ESRD), has become a major health problem worldwide [1]. In recent years, billions of dollars have been charged to the National Health Insurance system, and the cost has continued to rise [2]. CKD is essential to the overall morbidity and mortality due to non-communicable diseases [3]. CKD prevalence has increased in recent years, with an estimated 700 million people living with CKD and 1.2 million deaths worldwide in 2017 [3]. Approximately 13.3 million people, accounting for 13% of the Japanese adult population, are estimated to have CKD [4]. Cardiovascular disease (CVD) is the leading cause of morbidity and mortality in patients with CKD [5]. The prevalence of CVD in patients with CKD is almost 70%, nearly double the prevalence of CVD in the non-CKD populations [6]. Therefore, studying the risk factors that may lead to the damage and deterioration of renal function has become the top priority in preventing and treating kidney diseases.

Anemia is one of the most common complications of CKD, and the reported prevalence of anemia in patients with CKD ranges from 8.4% in stage 1 to 53.4% in stage 5 [7]. Anemia in CKD is associated with sleep disturbance, cognitive impairment, cardiovascular comorbidities, CKD progression, and higher mortality [8–11]. It has been well demonstrated among non-dialysis CKD patients, that anemia may be a risk factor for progression of kidney dysfunction not only to end-stage kidney disease (ESKD) or decrease of 50% of eGFR, but also to a faster decline of eGFR, a more significant renal outcome [12–18]. Hemoglobin concentration is related to the risk of cardiovascular disease and mortality in patients with and without diabetes [19–21]. Some studies suggest that low hemoglobin concentrations may be associated with an increased risk of poor kidney disease outcomes in people with type 2 diabetes [22, 23] and IgA nephropathy [24]. However, elevated hemoglobin levels have been implicated in a higher risk of mortality and cardiovascular events [25, 26] and CKD progression [27]. Thus, it seems as if there is a possibility of a non-linear relationship between hemoglobin and CKD progression. In addition, there are still few studies examining the relationship between specific hemoglobin (Hb) levels and renal prognosis and renal function decline simultaneously. Since CKD includes diabetic nephropathy, IgA nephropathy, interstitial nephritis, hereditary nephropathy, and other etiologies, the relationship between hemoglobin level and CKD progression in patients with CKD of different etiologies is still unclear. Therefore, the relationship between hemoglobin and CKD progression still needs further study. This study aimed to identify the linear and non-linear relationship between hemoglobin at baseline and renal prognosis and renal function decline in patients with CKD. On that account, a cohort study was designed to observe the relationship between Hb and CKD progression in the Japanese population with stage G2-G5 CKD.

## Methods

### Study design

This study used a prospective cohort study design. Data were obtained from the research on chronic kidney disease outcomes in treatment and epidemiology (CKD-ROUTE), a prospective, observational cohort study of a representative Japanese population with stage G2-G5 CKD. The stage of CKD was defined based on Kidney disease: improving global outcomes (KDIGO) classification [28]. Details of the design in the study have been reported previously [29–31]. More than 1,000 participants participated in Tokyo Medical and Dental University Hospital, and its 15 affiliated hospitals were enrolled [29]. We set hemoglobin at baseline as the target-independent variable and renal prognosis (renal composite endpoint, initiation of dialysis during follow-up or 50% decline in eGFR from baseline) and renal function decline(annual eGFR decline) as the dependent variable.

### Data source

We downloaded the raw data freely from the DATADRYAD database provided by Iimori S et al.. From: Prognosis of chronic kidney disease with normal-range proteinuria: The CKD-ROUTE study. *PLOS ONE* 2018, 13(1):e190493 [29]. Dryad Digital Repository. Dryad (https://datadryad.org/stash) data package (https://doi.org/10.5061/dryad.kq23s). Under Dryad's terms of service, researchers could use this data for secondary analyses without violating authors' rights.

### Study population

**Fig. 1:**
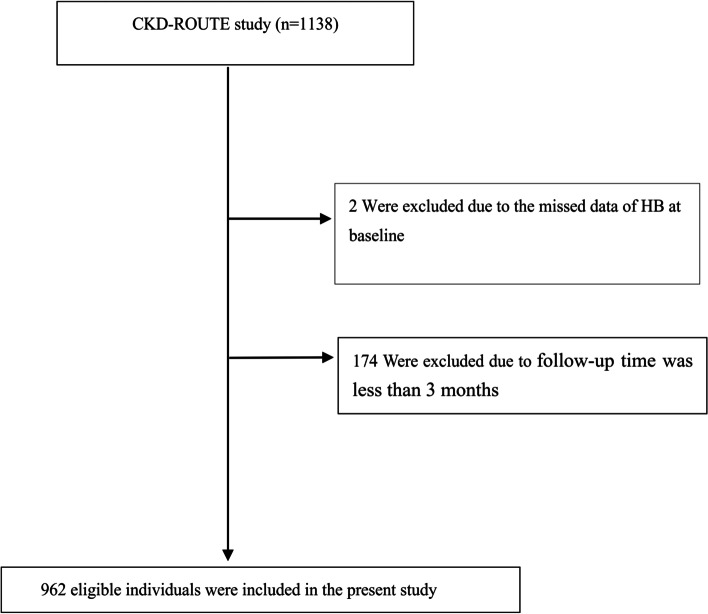
Flowchart of study participants. Figure 1 showed the inclusion of patients. 1,138 patients were assessed for eligibility in the original study. We excluded patients with missing values of Hb(*n* = 2), and follow-up time was less than 3 months (*n* = 174). The final analysis included 962 subjects in the present study

Because the most frequent bias was selection bias, which could lead to an over/underestimation of the obtained results. To minimize selection bias, the participants were collected non-selectively and consecutively in the Japanese population with stage G2-G5 CKD. Patients with stage 5 CKD were included in this study because a recent study showed that 35% of patients with stage 5 CKD did not receive renal replacement therapy during the 3-year observation period [32]. New patients over 20 years of age who presented or were referred for treatment but not dialysis between October 2010 and December 2011 were recruited. Patients with transplant recipients, malignancy, and/or active gastrointestinal bleeding; those who did not provide informed consent were excluded. Finally, 1,138 patients were assessed for eligibility in the original study [29]. We excluded patients with missing values of Hb (*n* = 2), and follow-up time was less than 3 months (*n* = 174). The final analysis included 962 subjects in the present study (see flowchart for details in Fig. 1).

In this study, participants' identifying information was encoded into untraceable codes to alleviate potential privacy concerns. Clinical data were extracted from the hospital's electronic medical record system. This research was conducted under the approval of the Ethics Committee in Tokyo Medical and Dental University Hospital and its 15 affiliated hospitals. All participants involved in this study have signed informed consent after explaining the study [29]. All methods were performed in accordance with the relevant guidelines and regulations by including a statement in the Declarations section.

### Variables

#### Hemoglobin

We obtained the hemoglobin information at baseline and recorded it as a continuous variable. Blood samples were collected to measure hemoglobin [31]. According to Japanese CKD guidelines, anemia was defined as hemoglobin level < 10 g/dL because the target Hb level for anemia treatment was 10–12 g/dL [33, 34].

#### Definitions of outcomes

The follow-up was the time from the date of baseline to the follow-up serum creatinine measurements (range 6–39 months). Outcomes(CKD progression) were defined, as in previously published work from the eGFR study [29, 35, 36], as follows: 1) Renal function decline: the annual eGFR decline (mL/min/1.73m^2^/year), which was calculated as the slope of the linear least-squares plot of eGFR values. 2) Renal prognosis: renal composite endpoint was defined as either initiation of dialysis during or 50% decline in eGFR from baseline.

Participants have been followed up at 6-month intervals. The total observation period was about 3 years after enrollment or until the occurrence of renal composite endpoint, time of death, withdrawal from the study, or withdrawal of informed consent.

#### Covariates

The covariates in this study were selected based on our clinical experience, original studies, and other studies investigating risk factors for CKD progression. Based on the above principles, wherefore, the following variables were used as covariates: (1) continuous variables: body mass index (BMI), age, urinary protein-to-creatinine ratio(UPCR), systolic blood pressure (SBP), estimated glomerular filtration rate (eGFR), and serum albumin(ALB); (2) categorical variables: gender, diabetes, urinary occult blood, history of CVD, hypertension, anti-hypertensive therapy including angiotensin receptor blockers(ARB), calcium channel blocker, angiotensin-converting enzyme inhibitors(ACEI), and diuretics.

Medical history, lifestyle behaviors (ability to self-feed), and current medications were recorded at enrollment. BMI was calculated from the body height and weight obtained by anthropometric measurements. BP was measured using a standard sphygmomanometer. Urine and blood samples were collected to measure creatinine, albumin, urinary occult blood, urinary protein, and urinary creatinine [29, 31]. The eGFR was calculated by the following diet modification in the renal disease equation modified for Japanese subjects [37]: eGFR = 194 × serum creatinine ^−1.094^ × age ^−0.287^ (if female, × 0.739). UPCR was measured because urine albumin was not routinely measured due to the Japanese health insurance system and was classified as: Optimal, UPCR < 0.15 g/gCr (gram per gram creatinine); High, UPCR 0.15–0.49 g/gCr; and very high, UPCR ≥ 0.5 g/gCr [31]. Low BMI (< 23.5 kg/m2) and low ALB level (< 4 g/dL) were defined as cut-off values (19).

### Definition of diabetes, hypertension, cardiovascular disease, and etiology of kidney disease

Hypertension was defined as SBP at least 140 mmHg or DBP at least 90 mmHg or clinician-diagnosed hypertension, or currently on anti-hypertensive medication [29]. Diabetes mellitus was defined as HbA1c ≥ 6.5% (National Glycohemoglobin Standardization Program (NGSP)method) or antidiabetic therapy history [29, 31]. The etiology of CKD in each patient was determined by the physician who treated the patient at the time of enrollment, according to the patient's clinical characteristics and findings, past medical history, and histological findings of renal biopsy specimens [29, 31].

CVD was defined as having a history of coronary heart disease (including myocardial infarction, angina pectoris, coronary revascularization), peripheral arterial disease, congestive heart failure, or stroke (cerebral infarction, transient ischemic attack, subarachnoid hemorrhage, or cerebral hemorrhage) [29].

### Statistical analysis

We stratified the participants by quintiles of the Hb level. Mean (standard deviation) (Gaussian distribution) or median(interquartile ranges)(Skewed distribution) were indicated for continuous variables, and frequencies and percentages were presented for categorical variables. One-Way ANOVA test (normal distribution), χ2 (categorical variables), or Kruskal-Whallis H test (skewed distribution) were used to detect the differences among different Hb groups. Person-years of follow-up were calculated from the baseline interview to the date of the renal composite endpoint event or the last date of the follow-up interview, whichever came first [38]. Incidence rates are expressed as cumulative incidence and person-year incidence [39]. Survival estimates and time-to-renal composite endpoint variables were computed using the Kaplan–Meier method. A log-rank test compared the Kaplan–Meier probability of renal composite endpoint-free survival among Hb groups [40].

To examine the link of Hb on renal prognosis and renal function decline, we constructed three models using univariate and multivariate Cox proportional-hazards and linear regression model, including the non-adjusted model (crude model: no covariates were adjusted), minimally-adjusted model (model I: only sociodemographic variables were adjusted, including gender, SBP, age, hypertension, BMI, history of CVD, diabetes,) and fully-adjusted model (model II: covariates presented in Table 1 were adjusted, including age, gender, BMI, SBP, UPCR, diabetes, eGFR, hypertension, ALB, urinary occult blood, use of RAAS inhibitor, history of CVD, use of CCB, use of diuretics). Effect sizes(HR and β) with 95% confidence intervals were recorded. We adjusted them when the covariances were added to the model and the HR or β changed by 10% or greater [41]. In a more standard Cox model that predicted only renal prognosis, both people would be censored out from the analysis at the time of death. Information about the risk of death competition was ignored. Competitive risk regression models take into account information from competitive risks and reweight renal composite endpoint risks based on competitive outcomes [42]. Therefore, competing risks multivariate Cox’s regression was performed, as described by Fine and Gray, with mortality as the competing risk for renal composite endpoint events [43, 44]. The results were expressed as a subdistribution HR (SHR) with 95% confidence intervals(CIs).

**Table 1:** Baseline characteristics of all the patients at enrollment (*n* = 962)

HB	Q1 (< 10.4)	Q2(10.4–12.0)	Q3(12.0–13.6)	Q4(≥ 13.6)	*P*-value
**Participants**	231	232	247	252	
**Age(years)**	71.54 ± 12.13	69.02 ± 13.27	67.03 ± 13.00	62.30 ± 14.03	< 0.001
**SBP(mmHg)**	140.81 ± 21.86	140.14 ± 22.19	136.89 ± 20.74	139.83 ± 22.53	0.206
**BMI(kg/m**^2^**)**	23.15 ± 4.46	23.78 ± 4.05	23.54 ± 3.91	24.50 ± 3.78	0.002
**HB(g/dL)**	9.19 ± 0.94	11.14 ± 0.45	12.76 ± 0.46	14.84 ± 0.99	< 0.001
**ALB(g/dL)**	3.53 ± 0.61	3.77 ± 0.54	4.01 ± 0.52	4.15 ± 0.56	< 0.001
**Scr(mg/dL)**	2.65 (1.91–3.99)	2.08 (1.50–2.92)	1.43 (1.10–1.96)	1.25 (1.06–1.55)	< 0.001
**eGFR (ml/min per 1.73 m**^2^**)**	19.83 ± 12.94	25.93 ± 13.25	37.53 ± 16.00	47.28 ± 15.64	< 0.001
**UPCR (g/gCr)**	1.75 (0.53–4.50)	1.22 (0.21–3.15)	0.28 (0.07–1.57)	0.27 (0.05–1.07)	< 0.001
**Gender**					< 0.001
**Male**	126 (54.55%)	148 (63.79%)	171 (69.23%)	225 (89.29%)	
**Female**	105 (45.45%)	84 (36.21%)	76 (30.77%)	27 (10.71%)	
**Etiology of CKD**					< 0.001
**Diabetic nephropathy, n(%)**	93 (40.26%)	74 (31.90%)	45 (18.22%)	32 (12.70%)	
**Nephrosclerosis, n (%)**	79 (34.20%)	84 (36.21%)	114 (46.15%)	108 (42.86%)	
**Glomerulonephritis, n (%)**	30 (12.99%)	40 (17.24%)	43 (17.41%)	65 (25.79%)	
**Other, n (%)**	29 (12.55%)	34 (14.66%)	45 (18.22%)	47 (18.65%)	
**Urinary occult blood, n(%)**	71 (30.74%)	101 (43.53%)	70 (28.34%)	68 (26.98%)	< 0.001
**Hypertension, n (%)**	220 (95.24%)	217 (93.53%)	220 (89.07%)	209 (82.94%)	< 0.001
**History of CVD, n (%)**	83 (35.93%)	70 (30.17%)	56 (22.67%)	49 (19.44%)	< 0.001
**Diabetes, n (%)**	112 (48.48%)	97 (41.81%)	90 (36.44%)	65 (25.79%)	< 0.001
**Use of RAAS inhibitor, n(%)**	156 (67.53%)	175 (75.43%)	160 (64.78%)	135 (53.57%)	< 0.001
**Use of calcium channel blocker, n (%)**	127 (54.98%)	121 (52.16%)	117 (47.37%)	94 (37.30%)	< 0.001
**Use of diuretics, n (%)**	123 (53.25%)	81 (34.91%)	60 (24.29%)	48 (19.05%)	< 0.001

Continuous variables are presented as mean ± standard deviation and median with interquartile ranges. Categorical data are presented as numbers and percentages

*Abbreviations*: BMI Body mass index, *Scr* Serum creatinine, *SBP* Systolic blood pressure, *HB* Hemoglobin, *ALB* Serum albumin, *CVD* Cardiovascular disease, *CKD* Chronic kidney disease, *eGFR* estimated glomerular filtration rate, *RAAS* Renin–angiotensin–aldosterone system, *UPCR* Urinary protein/creatinine ratio, *g/gCr* gram per gram creatinine

Since standard Cox proportional hazards regression models are often suspected of being incapable of handling non-linear models, on that account, non-linearity between hemoglobin and the renal prognosis was addressed using Cox proportional hazards regression model with cubic spline functions and the smooth curve fitting (penalized spline method) [45]. At the same time, a generalized additive model (GAM) and smooth curve fitting (penalized spline method) was conducted to explore the exact shape of the curve between ALB and renal function decline [46]. If non-linearity was detected, we first calculated the inflection point using a recursive algorithm and then constructed a two-piecewise Cox proportional-hazards regression model and two-piecewise linear regression model on both sides of the inflection point. A log-likelihood ratio test was used to determine the most appropriate model describing the association between ALB and renal prognosis and renal function decline [47].

The subgroup analyses were performed using a stratified Cox proportional-hazards regression model and a linear regression model across various subgroups (gender, age, BMI, ALB, urinary occult blood, SBP, UPCR, etiology of CKD, history of CVD, diabetes, hypertension, use of calcium channel blocker, use of diuretics and use of RAAS inhibitor). Firstly, we converted the continuous variable age (< 60, ≥ 60 years) [48], BMI (< 23.5, ≥ 23.5 kg/m^2^), SBP(< 140, ≥ 140 mmHg), UPCR(< 0.5, ≥ 0.5 g/gCr), ALB(< 4, ≥ 4 g/dL) to a categorical variable based on the clinical cut point. Secondly, in addition to the stratification factor itself, we adjusted each stratification for all factors (age, gender, BMI, SBP, UPCR, history of CVD, eGFR, hypertension, ALB, urinary occult blood, diabetes, use of RAAS inhibitor, use of calcium channel blocker, and use of diuretics). Lastly, tests for interaction were performed with the likelihood ratio test of models with and without interaction terms [49, 50].

The number of participants with missing data of SBP, BMI, ALB, urinary occult blood, and UPCR was 15(1.56%), 103(10.71%), 8(0.83%), 10(1.04%), and 66(6.86%), respectively. In order to maximize the use of participants' data and reduce potential bias resulting from missing data, multiple imputations were used to handle the missing data of covariants [51]. The imputation model included age, BMI, gender, SBP, etiology of CKD, diabetes, UPCR, hypertension, Scr, eGFR, ALB, history of CVD, urinary occult blood, use of RAAS inhibitor, use of calcium channel blocker, and use of diuretics. Missing data analysis procedures use missing-at-random (MAR) assumptions [52].

To test the robustness of our results, we performed a sensitivity analysis [53]. We transformed hemoglobin into a categorical variable according to quartiles. We calculated P for the trend to validate the results for hemoglobin as a continuous variable and to examine the possibility of non-linearity. As the risk of CKD progression was obviously increased in patients with massive proteinuria [54] and decreased renal function at baseline [55]. Therefore, when exploring the association between Hb and renal prognosis and renal function decline in other sensitivity analyses, we excluded participants with UPCR > 0.5 g/gCr or baseline eGFR < 15 ml/min per 1.73 m^2^. Besides, we also used a GAM to insert the continuity covariate into the equation as a curve to ensure the robustness of the results [56]. Additionally, we explored the potential for unmeasured confounding between the Hb and renal prognosis and renal function decline by calculating E-values [57]. All results were written according to the STROBE statement [41].

Modeling was performed with the statistical software packages R (http://www.R-project.org, The R Foundation) and EmpowerStats (http://www.empowerstats.com, X&Y Solutions, Inc, Boston, MA). *P* values less than 0.05 (two-sided) were considered statistically significant.

## Results

### Baseline characteristics of participants

Table 1 provided the baseline demographic and clinical characteristics of participants included in the study. The population at baseline, of whom 69.65% were male, had a mean age of 67.35 ± (13.56) years. The mean baseline Hb and eGFR were 12.06 ± 2.21 g/dL and 33.04 ± 18.01 ml/min per 1.73 m^2^. The annual decline in eGFR was 2.09 mL/min/1.73 m^2^/year. The incidence of renal composite endpoint was 26.19% (252/962). We assigned participants into subgroups using Hb quartiles (< 10.4, 10.4–12.0, 12.0–13.6, ≥ 13.6). No statistical difference was found in baseline characteristics in terms of SBP in different groups of hemoglobin(quartile) (*P*-value > 0.05). When we set the Q1(Hb < 10.4 g/dL) group as reference, the higher value or proportion of BMI, ALB, and eGFR were detected in the Q4 (Hb ≥ 13.6 g/dL) group, while the lower value and proportion of age, UCPR, Scr, females, hypertension, diabetic nephropathy, urinary occult blood, use of RAAS inhibitor, diabetes, use of calcium channel blocker, history of CVD, and use of diuretics were observed.

**Fig. 2:**
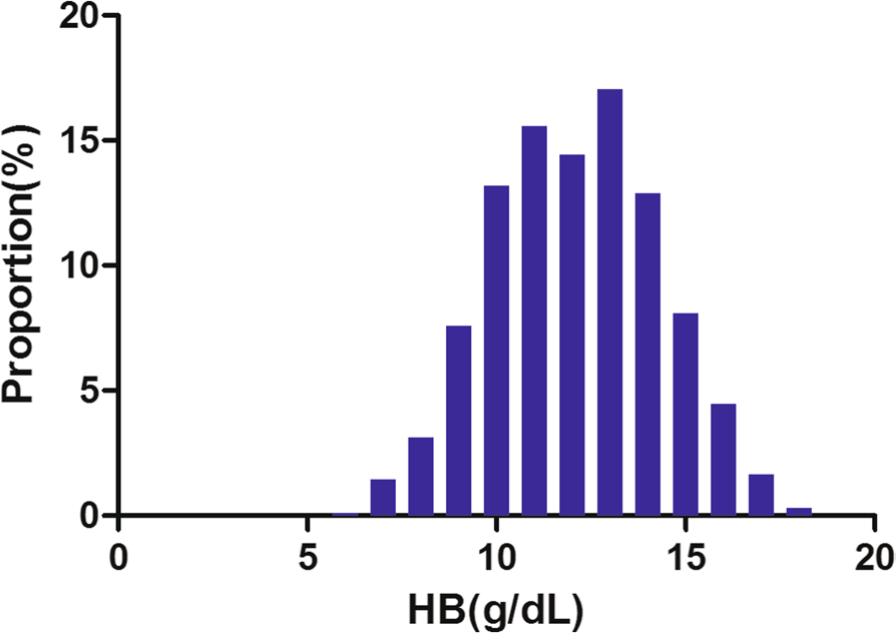
Distribution of hemoglobin. Figure 2. It presented a normal hemoglobin distribution while being in the range from 5.9 to 18.0 g/dL, with an average of 12.1 g/dL

**Fig. 3:**
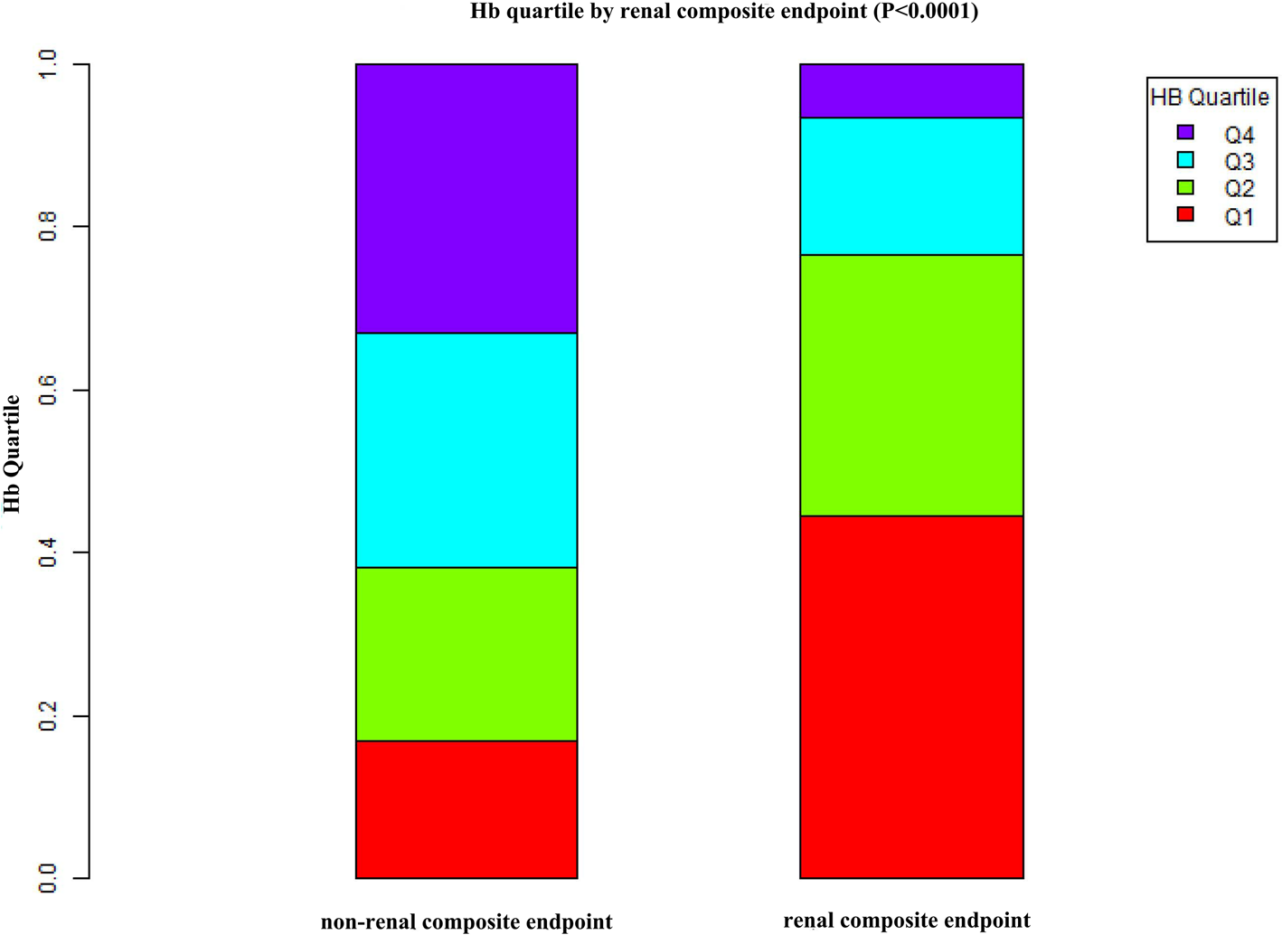
Data visualization of hemoglobin of all participants from the renal composite endpoint and non- renal composite endpoint groups. Figure 3 indicated that the distribution level of Hb in the renal composite endpoint group was lower. In contrast, the Hb level in the non-renal composite endpoint group was relatively higher

**Fig. 4:**
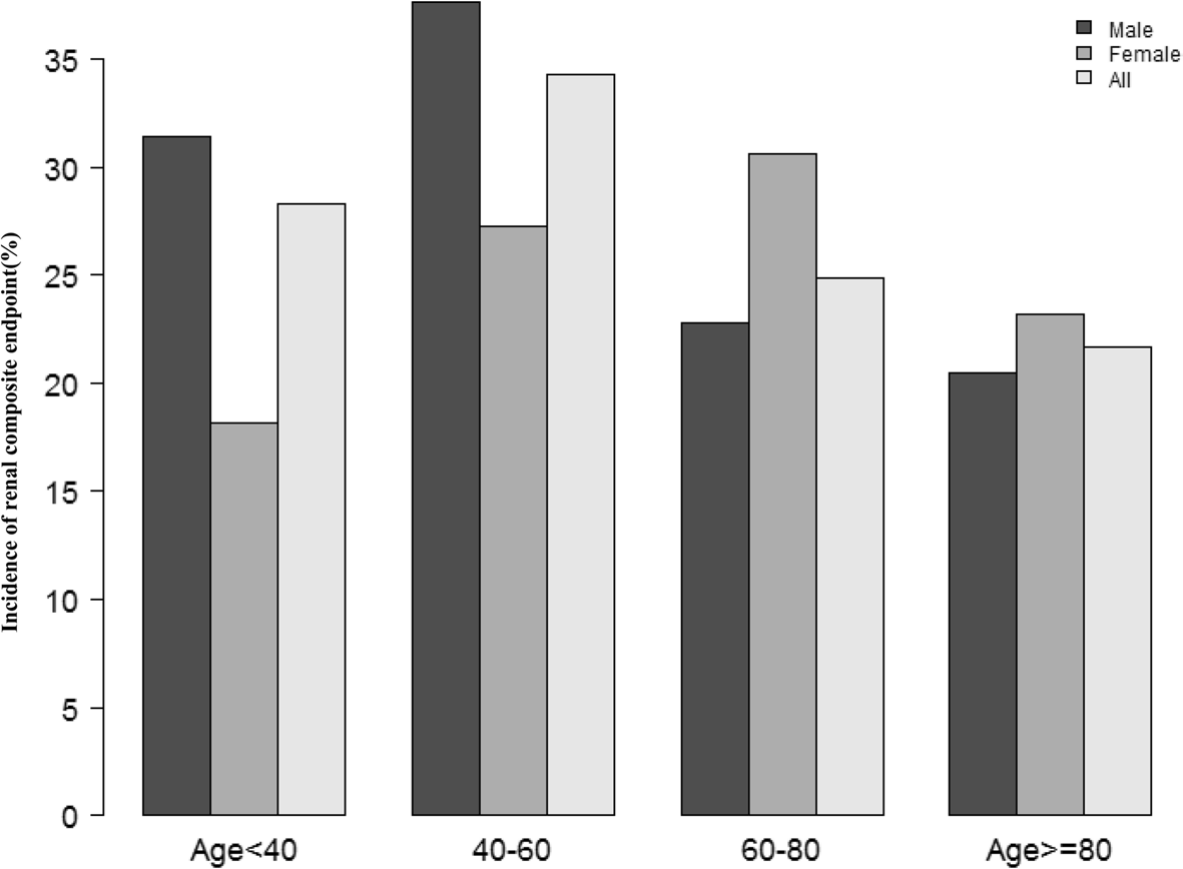
The renal composite endpoint incidence rate of age stratification by 20 intervals. Figure 4 showed that in age stratification by 20 intervals, when age < 60, male subjects had a higher incidence of renal composite endpoint than female subjects. In contrast, when age > 60, male subjects had a lower incidence of renal composite endpoint than female subjects

Figure 2 showed the distribution of Hb levels. It presented a normal distribution while being in the range from 5.9 to 18.0 g/dL, with an average of 12.1 g/dL. Participants were divided into two groups based on whether they experienced renal composite endpoint or not. The Hb values in the two groups were shown in Fig. 3. The results indicated that the distribution level of Hb in the renal composite endpoint group was lower. In contrast, the Hb level in the non-renal composite endpoint group was relatively higher. In age stratification by 20 intervals, when age < 60, male subjects had a higher incidence of renal composite endpoint than female subjects (Fig. 4). In contrast, when age > 60, male subjects had a lower incidence of renal composite endpoint than female subjects.

### The incidence rate of the renal composite endpoint

**Fig. 5:**
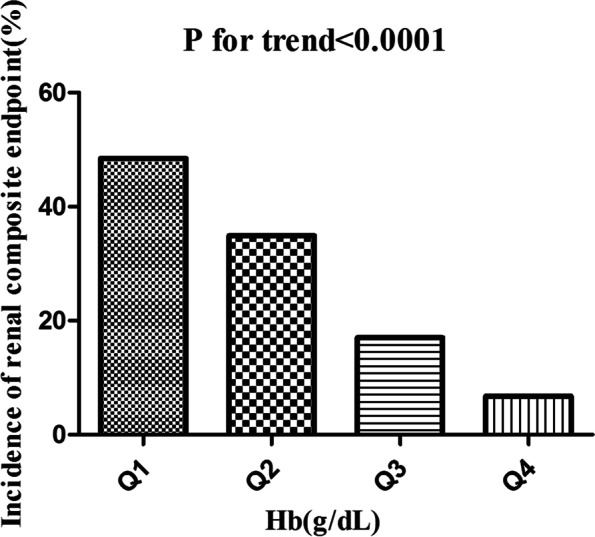
Incidence of renal composite endpoint according to the quintiles of hemoglobin. Figure 5. Compared with the lowest Hb group, participants with a high Hb had a lower incidence of the renal composite endpoint (*p* < 0.0001 for trend)

Table 2 revealed that 252(26.2%) patients developed renal composite endpoint in total during a median follow-up time of 33.5 months. The total cumulative incidence rate of all patients was 0.99 per 100 person-years. Specifically, the cumulative incidence rates of the four Hb groups were 2.33, 1.37, 0.58, and 0.23 per 100 person-years, respectively. The incidence of total renal composite endpoint and each Hb group was 26.20%(23.41–28.98%), 48.48%(41.99–54.98%), 34.91%(28.73–41.09%), 17.00%(12.29–21.72%), and 6.75%(3.63–9.86%), respectively. Compared with the lowest Hb group, participants with a high Hb had a lower incidence of the renal composite endpoint ( *p* < 0.0001 for trend) (Fig. 5).

**Table 2:** Incidence rate of the renal composite endpoint

HB	Participants(n)	Renal composite endpoint events(n)	Incidence rate(95% CI)(%)	Cumulative incidence( Per 100 person-year)
**Total**	962	252	26.20(23.41–28.98)	0.99
**Q1**	231	112	48.48(41.99–54.98)	2.33
**Q2**	232	81	34.91(28.73–41.09)	1.37
**Q3**	247	42	17.00(12.29–21.72)	0.58
**Q4**	252	17	6.75(3.63–9.86)	0.23
**P for trend**			< 0.0001

### The results of univariate analyses using Cox proportional-hazards regression model

The univariate analysis was conducted on the available data, showing that the factors in terms of age, gender, history of CVD, and BMI were not related to the renal composite endpoint (All *P*-value > 0.05), but SBP (HR = 1.017, 95%CI 1.012 to 1.022), Scr (HR = 1.449, 95%CI 1.396 to 1.505), urinary occult blood (HR = 1.658, 95%CI 1.290 to 2.130), UPCR (HR = 1.208, 95%CI 1.183 to 1.234), hypertension (HR = 4.070, 95%CI 1.920 to 8.627), diabetes (HR = 2.699, 95%CI 2.101 to 3.466), use of RAAS inhibitor (HR = 1.727, 95%CI 1.293 to 2.308), use of calcium channel blocker (HR = 1.724, 95%CI 1.338 to 2.221), and use of diuretics (HR = 2.197, 95%CI 1.715 to 2.815) were positively linked to the renal composite endpoint. And Hb (HR = 0.691, 95%CI 0.651 to 0.733), eGFR (HR = 0.917, 95%CI 0.906 to 0.929), and ALB (HR = 0.349, 95%CI 0.299 to 0.407) were negatively connected with renal composite endpoint (See Table 3 for detail). We also found that patients with primary onset diabetic nephropathy had a high risk of the renal composite endpoint.

**Table 3:** The results of univariate analysis for renal prognosis

Variable	Statistics	Effect size HR(95%CI)	*P* value
**Age(years)**	67.352 ± 13.558	0.992 (0.983, 1.001)	0.07672
**Gender**			
**Male**	670 (69.647%)	1.0	
**Female**	292 (30.353%)	1.064 (0.816, 1.385)	0.64813
**Etiology of CKD**			
**Diabetic nephropathy,**	244 (25.364%)	1.0	
**Nephrosclerosis,**	385 (40.021%)	0.185 (0.136, 0.254)	< 0.00001
**Glomerulonephritis**	178 (18.503%)	0.295 (0.208, 0.419)	< 0.00001
**Other**	155 (16.112%)	0.174 (0.110, 0.277)	< 0.00001
**BMI(kg/m2)**	23.756 ± 4.072	1.027 (0.995, 1.059)	0.09929
**SBP(mmHg)**	139.388 ± 21.853	1.017 (1.012, 1.022)	< 0.00001
**HB(g/dL)**	12.055 ± 2.213	0.691 (0.651, 0.733)	< 0.00001
**Scr(mg/dL)**	2.144 ± 1.469	1.449 (1.396, 1.505)	< 0.00001
**eGFR (ml/min per 1.73 m2)**	33.037 ± 18.007	0.917 (0.906, 0.929)	< 0.00001
**ALB(g/dL)**	3.874 ± 0.605	0.349 (0.299, 0.407)	< 0.00001
**Urinary occult blood**			
**No**	652 (67.775%)	1.0	
**Yes**	310 (32.225%)	1.658 (1.290, 2.130)	0.00008
**UPCR(g/gCr)**	2.037 ± 3.189	1.208 (1.183, 1.234)	< 0.00001
**Hypertension**			
**No**	96 (9.979%)	1.0	
**Yes**	866 (90.021%)	4.070 (1.920, 8.627)	0.00025
**History of CVD**			
**No**	704 (73.181%)	1.0	
**Yes**	258 (26.819%)	1.262 (0.962, 1.655)	0.09277
**Diabetes**			
**No**	598 (62.162%)	1.0	
**Yes**	364 (37.838%)	2.699 (2.101, 3.466)	< 0.00001
**Use of RAAS inhibitor**			
**No**	336 (34.927%)	1.0	
**Yes**	626 (65.073%)	1.727 (1.293, 2.308)	0.00022
**Use of calcium channel blocker**			
**No**	503 (52.287%)	1.0	
**Yes**	459 (47.713%)	1.724 (1.338, 2.221)	0.00002
**Use of diuretics**			
**No**	650 (67.568%)	1.0	
**Yes**	312 (32.432%)	2.197 (1.715, 2.815)	< 0.00001

**Fig. 6:**
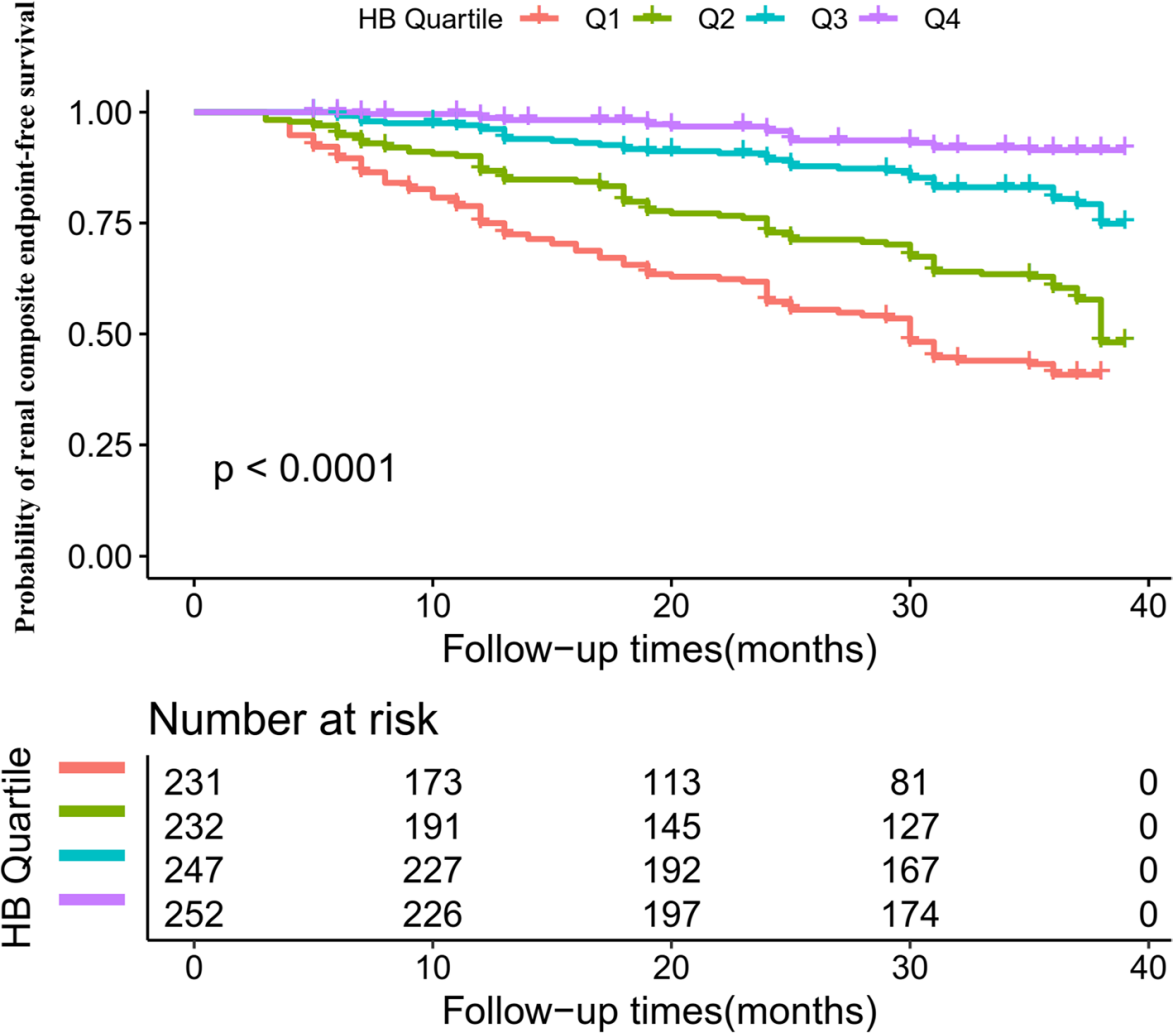
Kaplan–Meier event-free survival curve. Figure 6. Kaplan–Meier event-free survival curve. Kaplan–Meier analysis of incident renal composite endpoint-free survival based on Hb groups (log-rank, *P* < 0.0001)

Kaplan–Meier survival curves for renal composite endpoint-free survival probability stratified by the Hb group were shown in Fig. 6. The renal composite endpoint-free survival probability between Hb groups was significantly different (log-rank test, *p* < 0.0001). With the increased Hb, the probability of renal composite endpoint-free survival gradually increased, indicating the top group with the lowest renal composite endpoint risk.

### The results of multivariate analyses using Cox proportional-hazards regression model

The authors constructed three models using the Cox proportional-hazards regression model to investigate the relationship between hemoglobin and renal composite endpoint. In the unadjusted model, an increase of 1 g/dL of hemoglobin was associated with a 31% decrease in risk of the renal composite endpoint (HR = 0.691, 95%CI 0.651 to 0.733). The distribution of confidence intervals indicated that the connection between hemoglobin and renal composite endpoint obtained by the model was reliable. In the minimally-adjusted model, when we only adjusted for demographic variables, each additional g/dL of hemoglobin increase could lead to the risk of the renal composite endpoint decreasing by 32% (HR = 0.681, 95%CI 0.640 to 0.725). The findings on the connection between hemoglobin and renal composite endpoint obtained from the model were statistically significant. In the fully adjusted model, each additional g/dL of hemoglobin was accompanied by a 16% decrease in renal composite endpoint (HR = 0.836, 95%CI 0.770 to 0.907). The results were statistically significant (Table 4).

**Table 4:** Relationship between HB and the renal composite endpoint in different models

**Exposure**	Crude model (HR,95%CI, P)	Model I(HR,95%CI, P)	Model II (HR,95%CI, P)	Model III (HR,95%CI, P)
**HB**	0.691 (0.651, 0.733) < 0.00001	0.681 (0.640, 0.725) < 0.00001	0.836 (0.770, 0.907) 0.00002	0.863 (0.790, 0.944) 0.00123
**HB Quartile**				
**Q1**	Ref	Ref	Ref	Ref
**Q2**	0.569 (0.427, 0.758) 0.00012	0.572 (0.426, 0.769) 0.00022	0.713 (0.518, 0.981) 0.03764	0.978 (0.697, 1.373) 0.89912
**Q3**	0.237 (0.166, 0.338) < 0.00001	0.241 (0.167, 0.349) < 0.00001	0.522 (0.348, 0.783) 0.00168	0.743 (0.483, 1.142) 0.17528
**Q4**	0.093 (0.056, 0.154) < 0.00001	0.082 (0.048, 0.139) < 0.00001	0.332 (0.181, 0.610) 0.00038	0.382 (0.203, 0.719) 0.00286
**P for trend**	< 0.00001	< 0.00001	0.00005	0.00669

Crude model: we did not adjust other covariants

Model I: we adjust age, gender, BMI, SBP, hypertension, diabetes, and history of CVD

Model II: we adjust age, gender, BMI, SBP, hypertension, diabetes, history of CVD, UPCR, eGFR, ALB, urinary occult blood, use of RAAS inhibitor, use of calcium channel blocker, use of diuretics

*CI* Confidence, *Ref* Reference

Model III: we adjust age(smooth), gender, BMI(smooth), SBP(smooth), hypertension, diabetes, history of CVD, UPCR(smooth), eGFR(smooth), ALB(smooth), urinary occult blood, use of RAAS inhibitor, use of calcium channel blocker, use of diuretics

*HR* Hazard ratios, *CI* Confidence, *Ref* Reference

### The results of competing risks multivariate Cox’s regression

When death was treated as a competing event, the competing analysis results were shown in Table 5. In the crude model, Hb showed a negative association with the renal composite endpoint (SHR = 0.69, 95% confidence interval (CI):0.65 to 0.73). The minimally adjusted model (adjusted gender, age, SBP, BMI, hypertension, diabetes, and history of CVD) did not show the apparent change (SHR:0.68, 95%CI: 0.64–0.72). In the fully adjusted model (model II) (adjusted gender, hypertension, age, diabetes, BMI, history of CVD, UPCR, SBP, eGFR, ALB, urinary occult blood, use of calcium channel blocker, use of RAAS inhibitor, and use of diuretics), we could also detect the connection (SHR = 0.84, 95%CI: 0.77 to 0.91). This result was similar to that when the competing risk of death was not considered.

**Table 5:** Relationship between HB and the renal composite endpoint in different models with competing risk of mortality

**Exposure**	Crude model (SHR,95%CI, P)	Model I(SHR,95%CI, P)	Model II (SHR,95%CI, P)
**HB**	0.69 (0.65, 0.73) < 0.0001	0.68 (0.64, 0.72) < 0.0001	0.84 (0.77, 0.91) < 0.0001
**HB Quartile**			
**Q1**	Ref	Ref	Ref
**Q2**	0.57(0.43, 0.76) 0.0001	0.57 (0.43, 0.77) 0.0002	0.72 (0.53, 0.99) 0.0462
**Q3**	0.24 (0.17, 0.34) < 0.0001	0.24 (0.17, 0.35) < 0.0001	0.53 (0.35, 0.79) 0.0019
**Q4**	0.09 (0.06, 0.15) < 0.0001	0.08 (0.05, 0.14) < 0.00001	0.34(0.18, 0.62) 0.0004
**P for trend**	< 0.0001	< 0.0001	< 0.0001

Crude model: we did not adjust other covariants

Model I: we adjust age, gender, BMI, SBP, hypertension, diabetes, and history of CVD

Model II: we adjust age, gender, BMI, SBP, hypertension, diabetes, history of CVD, UPCR, eGFR, ALB, urinary occult blood, use of RAAS inhibitor, use of calcium channel blocker, use of diuretics

*SHR* Subdistribution hazard ratios, *CI* Confidence, *Ref* Reference

### The results of multivariate analyses using linear regression model

We constructed three linear regression models to investigate the relationship between serum albumin and renal function decline. In the unadjusted model (crude model), an increase of 1 g/dL of Hb was associated with 0.298 mL/min/1.73 m^2^/year decreases in annual eGFR decline(β = -0.298, 95%CI -0.551 to -0.045). The distribution of confidence intervals indicated that the connection between Hb and the annual decline in eGFR obtained by the model was reliable. In the minimally-adjusted model(model I), when we only adjusted for demographic variables, each additional g/dL of Hb increase could lead to the annual eGFR decline decreasing 0.340 mL/min/1.73 m^2^/year (β = -0.340, 95%CI -0.620 to -0.061). The findings on the connection between Hb and renal function decline obtained from the model were statistically significant. In the fully adjusted model (model II), each additional g/dL of Hb was accompanied by a 0.436 mL/min/1.73 m^2^/year decrease in annual eGFR decline (β = -0.436, 95%CI -0.778 to -0.093). The results were statistically significant (Table 6).

**Table 6:** Relationship between Hb and renal function decline in different models

**Exposure**	Crude model (β,95%CI, P)	Model I (β,95%CI, P)	Model II (β,95%CI, P)	Model III (β,95%CI, P)
**Hb**	-0.298 (-0.551, -0.045) 0.02118	-0.340 (-0.620, -0.061) 0.01731	-0.436 (-0.778, -0.093) 0.01288	-0.428 (-0.777, -0.079) 0.01638
**Hb Quartile**				
**Q1**	Ref	Ref	Ref	Ref
**Q2**	0.767 (-0.847, 2.381) 0.35164	0.789 (-0.802, 2.381) 0.33126	0.745 (-0.876, 2.365) 0.36799	0.976 (-0.631, 2.583) 0.23427
**Q3**	-0.186 (-1.775, 1.404) 0.81898	0.205 (-1.414, 1.825) 0.80377	-0.163 (-1.949, 1.623) 0.85844	0.059 (-1.719, 1.837) 0.94813
**Q4**	-1.322 (-2.903, 0.260) 0.10184	-1.509 (-3.250, 0.232) 0.08964	-2.118 (-4.201, -0.034) 0.04661	-1.795 (-3.868, 0.278) 0.08996
**P for trend**	0.05020	0.07445	0.04345	0.06906

Crude model: we did not adjust other covariants

Model I: we adjust age, gender, BMI, SBP, hypertension, diabetes, history of CVD, and etiology of CKD

Model II: we adjust age, gender, BMI, SBP, hypertension, diabetes, etiology of CKD, history of CVD, UPCR, eGFR, ALB, urinary occult blood, use of RAAS inhibitor, use of calcium channel blocker, use of diuretics

*CI* Confidence, *Ref* Reference

Model III: we adjust age(smooth), gender, BMI(smooth), SBP(smooth), hypertension, etiology of CKD, diabetes, history of CVD, UPCR(smooth), eGFR(smooth), ALB(smooth), urinary occult blood, use of RAAS inhibitor, use of calcium channel blocker, use of diuretics

*CI* Confidence, *Ref* Reference

### Sensitivity analysis

To verify the robustness of our findings, a series of sensitivity analyses were performed. We first convert hemoglobin from a continuous variable to a categorical variable (according to quartile) and then put categorical-transformed hemoglobin back into the model. The results showed that after hemoglobin was transformed into a categorical variable, the trend of the effect sizes (HR, SHR, or β) in different groups is equidistant, and P for the trend is consistent with the result when hemoglobin is a continuous variable (Tables 4, 5 and 6).

In addition, we used a GAM to insert the continuity covariate into the equation as a curve. The result of Model III in Tables 4 and 6 showed this generally remained consistent with the fully adjusted model (HR = 0.863, 95%CI: 0.770–0.907, *P* = 0.00123) and (β = -0.428, 95%CI: -0.777 to -0.079, *P* = 0.01638). Besides, we generated an E-value to assess the sensitivity to unmeasured confounding. The E-values of HR and β were 1.52 and 4.64. The E-value was greater than the relative risk of unmeasured confounders and Hb, suggesting unmeasured or unknown confounders had little effect on the relationship between Hb and renal prognosis and renal function decline.

Furthermore, we excluded participants with UPCR > 0.5 g/gCr in other sensitivity analyses. The results suggested that after adjusting the confounding factors, Hb was also negatively associated with renal prognosis (HR = 0.622, 95% confidence interval (CI):0.474 to 0.815) and the annual eGFR decline(β = -0.201, 95%CI:-0.625 to 0.223) (Table 7). We also excluded participants with baseline eGFR < 15 ml/min per 1.73 m^2^ for sensitivity analyses. The results suggested that after adjusting gender, age, SBP, BMI, hypertension, diabetes, history of CVD, UPCR, eGFR, ALB, urinary occult blood, use of calcium channel blocker, use of RAAS inhibitor, and use of diuretics, Hb was still negatively associated with renal prognosis (HR = 0.837, 95% confidence interval (CI):0.757 to 0.926) and the annual eGFR decline(β = -0.408, 95%CI:-0.789 to -0.027) (Table 7). The results obtained from the sensitivity analyses indicated the well-robustness of our findings.

**Table 7:** Relationship between Hb and renal prognosis and renal function decline in different sensitivity analyses

Exposure	Model I(HR,95%CI, P)	Model II (β,95%CI, P)
**eGFR ≥ 15(ml/min per 1.73 m2)**		
Hb	0.837 (0.757, 0.926) 0.00053	-0.408 (-0.789, -0.027) 0.03608
Hb Quartile		
Q1	Ref	Ref
Q2	0.928 (0.586, 1.470) 0.75033	0.680 (-1.351, 2.711) 0.51203
Q3	0.600 (0.352, 1.021) 0.05944	-0.009 (-2.116, 2.097) 0.99313
Q4	0.380 (0.193, 0.748) 0.00508	-1.789 (-4.141, 0.563) 0.13641
P for trend	0.00195	0.07496
**UPCR < 0.5 g/gCr**		
Hb	0.622 (0.474, 0.815) 0.00058	-0.201 (-0.625, 0.223) 0.35305
Hb Quartile		
Q1	Ref	Ref
Q2	0.380 (0.140, 1.029) 0.05683	0.956 (-1.518, 3.429) 0.44930
Q3	0.172 (0.044, 0.678) 0.01188	0.014 (-2.370, 2.398) 0.99059
Q4	0.145 (0.022, 0.946) 0.04357	-0.396 (-3.061, 2.269) 0.77110 0.49540
P for trend	0.0066	

Model I was a sensitivity analysis of the relationship between Hb and renal prognosis. We adjusted age, gender, BMI, SBP, hypertension, diabetes, history of CVD, UPCR, eGFR, ALB, urinary occult blood, use of RAAS inhibitor, use of calcium channel blocker, use of diuretics

Model II was a sensitivity analysis of the relationship between Hb and kidney function decline. We adjusted age, gender, BMI, SBP, hypertension, diabetes, history of CVD, UPCR, eGFR, ALB, urinary occult blood, use of RAAS inhibitor, use of calcium channel blocker, use of diuretics

*HR* Harzard ratios, *CI* Confidence, *Ref* Reference

### The non-linear relationship

**Fig. 7:**
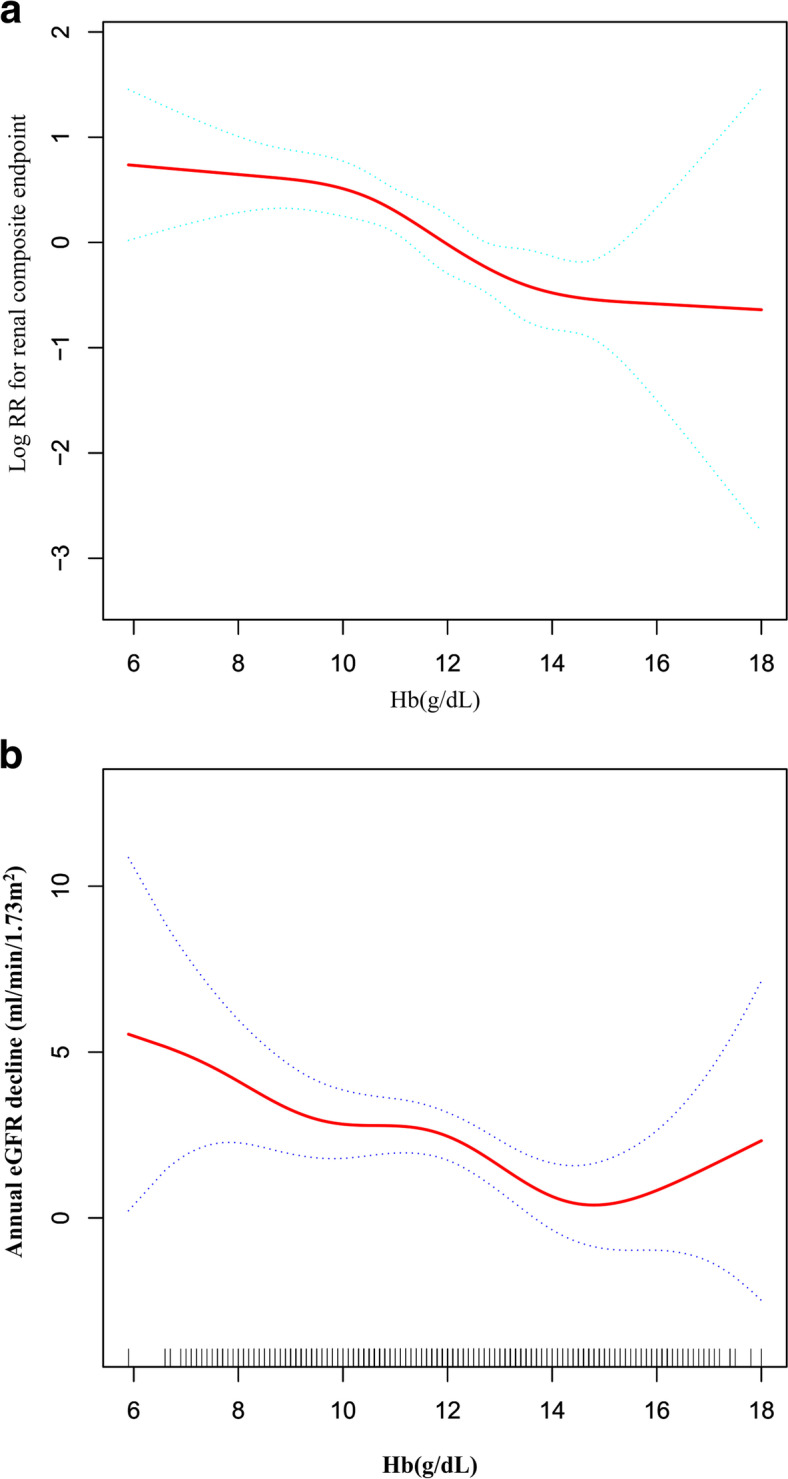
The non-linear relationship between hemoglobin and the risk of CKD progression. **A** We used a Cox proportional hazards regression model with cubic spline functions and smooth curve fitting (penalized spline method) to evaluate the relationship between Hb and renal prognosis. The result showed that the relationship between Hb and the renal prognosis was non-linear after adjusting for age, gender, BMI, SBP, hypertension, diabetes, history of CVD, UPCR, eGFR, ALB, urinary occult blood, use of RAAS inhibitor, use of calcium channel blocker and use of diuretics. **B** We also used a GAM and smooth curve fitting to evaluate the relationship between Hb and the annual decline in eGFR. The result showed that there was no non-linear relationship between hemoglobin and the annual decline in eGFR

Using a Cox proportional hazards regression model with cubic splines, we observed that the association between hemoglobin and the renal prognosis was non-linear (Fig. 7A). Therefore, we fit the data to a piecewise Cox regression model to obtain two distinct slopes. We also fit the data by a standard Cox regression model and obtained the best fit model by log-likelihood ratio test (Table 8). In this study, the log-likelihood ratio test had a *P*-value of 0.026, so ultimately we used a two-segment model to fit the association between hemoglobin and renal prognosis. We first obtained an inflection point of 8.6 g/dL through a recursive algorithm and then calculated the effect size (HR) and confidence intervals around the inflection point by a two-piece Cox proportional hazards regression model. On the left side of the inflection point, the HR and 95%CI were 1.257 (0.841, 1.878). On the right side of the inflection point, the HR and 95%CI were 0.789 (0.715, 0.870), respectively.

**Table 8:** The result of the two-piecewise Cox regression model and linear regression model

	**Renal prognosis (HR,95%CI, P)**
Fitting model by two-piecewise Cox regression	
Inflection point of Hb	8.6 g/dL
≤ 8.6 g/dL	1.257 (0.841, 1.878) 0.2650
> 8.6 g/dL	0.789 (0.715, 0.870) < 0.0001
P for log-likelihood ratio test	0.026
	**Renal function decline(β,95%CI, P)**
Fitting model by two-piecewise linear regression	
Inflection point of Hb	15.5 g/dL
≤ 15.5 g/dL	-0.551 (-0.920, -0.182) 0.0035
> 15.5 g/dL	1.527 (-0.830, 3.883) 0.2045
P for log-likelihood ratio test	0.096

*HR* Hazard ratios, *CI* Confidence interval

We adjusted age, gender, BMI, SBP, hypertension, diabetes, history of CVD, UPCR, eGFR, ALB, urinary occult blood, use of RAAS inhibitor, use of calcium channel blocker, and use of diuretics

We also used a GAM and smooth curve fitting to evaluate the relationship between Hb and the annual decline in eGFR (Fig. 7B). Although a seemingly non-linear relationship between Hb and the annual decline in eGFR could be found in Fig. 7B. However, we further found the *P*-value for the log-likelihood ratio test was not greater than 0.05. It indicated that there was no non-linear relationship between hemoglobin and the annual decline in eGFR (Table 8).

### The results of subgroup analyses

In all of the prespecified or exploratory subgroups evaluated (Table 9), there was no significant interaction in age, gender, urinary occult blood, BMI, etiology of CKD, use of RAAS inhibitor, history of CVD, diabetes, use of calcium channel blocker, and ALB. In contrast, significant interactions were detected in variables such as SBP, UPCR, hypertension, and the use of diuretics.

**Table 9:** Results of subgroup analysis and interaction analysis

Characteristic	participants	HR (95%CI) P for interacion	β(95%CI) P for interacion
Age(years)		0.6636	0.5920
< 60(years)	218	0.859 (0.747, 0.988)	-0.308 (-0.881, 0.264)
≥ 60(years)	744	0.829 (0.751, 0.914)	-0.466 (-0.831, -0.102)
Gender		0.8543	0.6664
Male	670	0.844 (0.766, 0.930)	-0.480 (-0.868, -0.092)
Female	292	0.831 (0.722, 0.956)	-0.304 (-1.026, 0.417)
Urinary occult blood		0.1975	0.5015
No	652	0.874 (0.786, 0.972)	-0.380 (-0.760, -0.001)
Yes	310	0.788 (0.697, 0.892)	-0.566 (-1.080, -0.052)
BMI(kg/m2)		0.5280	0.1642
< 23.5	492	0.825 (0.737, 0.924)	-0.715 (-1.168, -0.262)
≥ 23.5	470	0.866 (0.775, 0.968)	-0.304 (-0.752, 0.144)
SBP(mmHg)		0.0276	0.3823
< 140	514	0.741 (0.651, 0.843)	-0.317 (-0.740, 0.107)
≥ 140	448	0.871 (0.791, 0.959)	-0.536 (-0.961, -0.111)
UPCR (g/gCr)		0.0145	0.2201
< 0.5	458	0.683 (0.548, 0.851)	-0.217 (-0.742, 0.308)
≥ 0.5	504	0.903 (0.832, 0.981)	-0.637 (-1.075, -0.199)
Etiology of CKD		0.3714	0.9391
Diabetic nephropathy	44	0.781 (0.692, 0.881)	-0.498 (-1.049, 0.053)
Nephrosclerosis	85	0.943 (0.786, 1.130)	-0.391 (-0.846, 0.064)
Glomerulonephritis	178	0.799 (0.605, 1.055)	-0.566 (-1.227, 0.096)
Other	155	0.868 (0.613, 1.230)	-0.338 (-0.990, 0.314)
Use of RAAS inhibitor		0.9565	0.7207
No	336	0.830 (0.712, 0.967)	-0.496 (-0.974, -0.018)
Yes	626	0.834 (0.761, 0.913)	-0.404 (-0.789, -0.018)
Hypertension		0.0173	0.6052
No	96	0.435 (0.257, 0.734)	-0.716 (-1.657, 0.225)
Yes	866	0.842 (0.775, 0.915)	-0.457 (-0.823, -0.091)
History of CVD		0.0978	0.1901
No	704	0.862 (0.787, 0.944)	-0.323 (-0.706, 0.059)
Yes	258	0.755 (0.652, 0.874)	-0.694 (-1.212, -0.175)
Diabetes		0.7648	0.1948
No	598	0.819 (0.728, 0.922)	-0.592 (-1.005, -0.179)
Yes	364	0.837 (0.759, 0.923)	-0.208 (-0.697, 0.280)
Use of calcium channel blocker		0.2353	0.7644
No	503	0.791 (0.698, 0.897)	-0.474 (-0.901, -0.047)
Yes	459	0.869 (0.783, 0.965)	-0.398 (-0.821, 0.024)
Use of diuretics		0.0271	0.3428
No	650	0.903 (0.811, 1.005)	-0.615 (-1.010, -0.220)
Yes	312	0.761 (0.676, 0.856)	-0.329 (-0.854, 0.195)
ALB(g/dL)		0.8595	0.1032
< 4	465	0.837 (0.768, 0.911)	-0.716 (-1.129, -0.303)
≥ 4	497	0.852 (0.711, 1.021)	-0.280 (-0.718, 0.159)

Note 1:Above model adjusted for age, gender, BMI, SBP, hypertension, diabetes, history of CVD, UPCR, eGFR, ALB, urinary occult blood, use of RAAS inhibitor, use of calcium channel blocker, and use of diuretics

Note 2:In each case, the model is not adjusted for the stratification variable

Specifically, a stronger association between Hb and renal prognosis was observed in non-hypertensive (HR = 0.435,95%CI:0.257–0.734), SBP < 140 mmHg(HR = 0.741, 95%CI: 0.651–0.843), UPCR < 0.5 g/gCr (HR = 0.683,95%CI:0.548–0.851), and diuretic use patients (HR = 0.761,95%CI:0.676–0.856). In contrast, the weaker association was probed in hypertensive (HR = 0.842,95%CI:0.775–0.915) and non-diuretic use patients (HR = 0.903,95%CI:0.811–1.005) and in the participants with SBP ≥ 140 mmHg(HR = 0.871,95%CI:0.791–0.959), and UPCR ≥ 0.5 g/gCr(HR = 0.903,95%CI:0.832–0.981).

The results of subgroup analyses also indicated that the relationship of Hb with renal function decline was not affected by stratification variables.

## Discussion

In a recent retrospective research with multivariable-adjusted Cox proportional hazards models, Song et al. found that Hb was negatively associated with CKD progression in 265 patients with type 2 diabetes mellitus (HR = 0.65, 95%CI: 0.48–0.88, *P* = 0.0055) [23]. In another retrospective cohort study that included 4326 patients with IgA nephropathy, Oh, T R et al. found that baseline hemoglobin levels were also negatively associated with renal prognosis (HR = 0.871, 95%CI: 0.773–0.983, *P* = 0.025) [24]. However, in their further subgroup analyses, reduced serum hemoglobin was an independent risk factor for IgA nephropathy progression only in women(HR = 0.875, 95%CI: 0.768–0.998, *P* = 0.046), while in men, the HR and 95%CI were 0.937,0.858, 1.023, respectively. There was no statistically significant interaction of serum hemoglobin between men and women (P interaction = 0.177) [24]. In our research, having a medium sample size, the multivariable-adjusted Cox proportional hazards models showed a negative association between Hb and renal composite endpoint, consistent with those two studies. Besides, our subgroup analysis showed that the relationship between hemoglobin and renal composite endpoint was consistent in both males (HR = 0.844, 95%CI: 0.766–0.930, *P* = 0.0006) and females (HR = 0.831, 95%CI: 0.722–0.956, *P* = 0.0096). There was also no statistically significant interaction of hemoglobin between men and women (P interaction = 0.8543). The interaction of hemoglobin between men and women was also consistent with the findings of Oh, T R et al. [24]. Unlike them, our study found a statistically significant relationship between hemoglobin and CKD progression in both male and female patients. The inconsistent results might come from the following: (1) The study population was different. The studies of Oh, T R et al. focused on IgA nephropathy, while the participants in the present study were CKD patients with diabetic nephropathy, nephrosclerosis, glomerulonephritis, and other nephritis. (2) Compared with our research, their study did not consider the effect of hypertension, BMI, use of RAAS inhibitor, history of CVD, use of calcium channel blocker, urinary occult blood, and use of diuretics on the relationship between hemoglobin and CKD progression when adjusting. In addition, previous studies considered these variables in relation to CKD progression [58–65]. (4) This may be related to different baseline renal functions.

In the current study, we used a two-piecewise Cox regression model to clarify a non-linear relation between Hb and renal composite endpoint. The inflection point was 8.6 g/dL after adjusting for confounders. It showed that when Hb was above 8.6 g/dL, a 1 g/dL increase in Hb levels was associated with a 21.1% reduction in adjusted HR for the risk of the renal composite endpoint (HR = 0.789, 95%CI: 0.715–0.870). However, when Hb < 8.6 g/dL, a 1 unit increase in the Hb level was not associated with the adjusted HR of the renal composite endpoint (HR = 1.257, 95%CI: 0.841–1.878). The reason might be that other variables in the participant’s baseline also influenced CKD progression. It could be seen from Table S1 that compared with the Hb ≥ 8.6 g/dL, patients with Hb < 8.6 g/dL have generally higher age, Scr, UPCR levels, higher rates of diabetes, and use of diuretics. In contrast, patients generally had lower ALB and eGFR levels in the Hb < 8.6 g/dL group. However, the abnormality of the above indicators was closely related to the progress of CKD [54, 55, 65–68]. When Hb was less than 8.6 g/dL, due to the presence of these CKD progression risk factors, Hb had a relatively weak effect on the development of CKD progression. On the contrary, when HB was greater than 8.6 g/dL, the risk factors for CKD progressions, such as age, Scr, UPCR levels, higher rates of diabetes, and use of diuretics, were low. The impact on the occurrence of CKD progression was weakened; at this time, the effect of Hb was relatively enhanced.

Song and Oh, T R et al. also found a non-linear relationship between hemoglobin and CKD progression in patients with diabetes or IgA nephropathy. They found that when baseline hemoglobin was at 13.5–15.2 g/dL, the hemoglobin levels had the lowest log relative hazards of CKD progression [23, 24]. Considering factors such as CKD etiology, duration of follow-up, baseline eGFR, and hemoglobin levels, our non-linear results differ from those of their study. We also further explored the association of anemia and elevated hemoglobin with the renal composite endpoint in Tables S2 and S3. The results suggested that after adjusting for confounding variables, compared with patients with hemoglobin levels of 10–12 g/dL, those with a hemoglobin level below 10 g/dL had a 23% increased risk of the renal composite endpoint. In contrast, those with a hemoglobin greater than 12 g/dL had a 40% lower relative risk of the renal composite endpoint. Although no hemoglobin levels with the lowest log relative hazards were found in our research, according to the shape of the non-linear relationship between hemoglobin and renal composite endpoint and renal function decline, the change in the log relative hazards of renal composite endpoint and the annual eGFR decline was no longer evident as hemoglobin increased when hemoglobin was greater than 14 g/dL. This was basically consistent with the findings of Song and Oh, T R et al.

Our study has some strengths, and we listed them as follows. (1) Most covariates have complete information, with few missing. Multiple imputation was employed to handle missing data. This method could maximize statistical power and minimize potential bias caused by covariate information missing. (2) The robustness of this study was tested with a set of sensitivity analyses(target independent variable transformation, subgroup analysis, using a GAM to insert the continuity covariate into the equation as a curve, calculating E-values to explore the potential for unmeasured confounding, and reanalyzing the association between Hb and CKD progression after excluding participants with UPCR ≥ 0.5 g/gCr or eGFR < 15 ml/min per 1.73 m^2^) to ensure the reliability of the results. (4) We expounded on the non-linear relationship and found the inflection point. (5) Considering mortality as the competing risk for renal composite endpoint events, we performed competing risks multivariate Cox’s regression.

Our research has the following shortcomings and needs attention. First, the data was obtained from the study of the CKD-ROUTE in Japan, and the data has been screened by Soichiro Iimori et al. [29]. Therefore, we could not conclude whether our findings are suitable for people with other diseases or in a different race. This may further limit the generalizability of the results. However, we found that the association between Hb and CKD progression was stable in patients of different ages, gender, and etiologies by subgroup analysis. It also suggests that this study has some generalizable value to some extent. Second, since this was secondary data analysis, factors not measured in the original study could not be adjusted, such as blood lipid levels and diet status. We also could not evaluate the effect of iron status and usage of drugs, such as immunosuppressants and eritropoetin. However, we calculated E-value to quantify the potential impact of unmeasured confounders and found that unmeasured confounders were unlikely to explain the results. In the future, we can consider designing our studies or collaborating with other researchers to collect as many variables as possible, including information on blood lipid levels, diet status, iron status, and usage of drugs. Third, the attending doctor's diagnosis determined the etiology of CKD. Many patients did not undergo renal biopsy. Fourth, the present study only measured Hb, and other parameters at baseline did not consider changes in Hb over time. Fifth, because of the study's observational nature, this analysis only confirms that lower Hb values are associated with faster decline in renal function, but a clear cause-effect relationship cannot be drawn. Meanwhile, the evidence of the association between Hb level and renal progression does not allow per se to indicate or suggest therapeutic consequences in the management of anemia. The therapeutic implications might be verified by adequate RCT.

## Conclusion

This study demonstrates a negative and non-linear relationship between hemoglobin and CKD progression in Japanese CKD patients. There is a threshold effect between hemoglobin levels and renal composite endpoint. When hemoglobin level exceeds 8.6 g/dL, there is a significant negative association with renal composite endpoint riks. As hemoglobin is a common and easily available measurement in clinical activity, it is convenient and feasible to identify and treat patients at high risk of CKD progression.

## Supplementary Information

**Additional file 1:Table S1.** The Baseline Characteristics of participants on both sides of the inflection point. **Table S2.** The baseline characteristics of participants according to the clinical cut-off point for hemoglobin. **Table S3.** Relationship between Hb group and the renal composite endpoint in different models.

## Data Availability

Data can be downloaded from the ‘DATADRYAD’ database, which is a public database, (https://datadryad.org/stash) data package (https://doi.org/10.5061/dryad.kq23s).

## Abbreviations

Hb: Hemoglobin
UPCR: Urinary protein-to-creatinine ratio
CKD: Chronic kidney disease
CKD-ROUTE: Chronic kidney disease research of outcomes in treatment and epidemiology
KDIGO: Kidney Disease Improving Global Outcomes
BMI: Body mass index
ESRD: End-stage renal disease
SBP: Systolic blood pressure
Scr: Serum creatinine
DBP: Diastolic blood pressure
eGFR: Estimated glomerular filtration rate
ALB: Serum albumin
ARB: Angiotensin receptor blockers
MI: Myocardial infarction
CVD: Cardiovascular disease
ACEI: Angiotensin-converting enzyme inhibitors
CHF: Congestive heart failure
AP: Angina pectoris
PAD: Peripheral arterial disease
HR: Hazard ratios
CI: Confidence intervals
SHR: Subdistribution hazard ratios
