# Supplementary material for: Association between hemoglobin and chronic kidney disease progression: a secondary analysis of a prospective cohort study in Japanese patients
Source: BMC Nephrol. 2022 Aug 23;23:295. doi: 10.1186/s12882-022-02920-6 (PMC9400271; doi:10.1186/s12882-022-02920-6)
Supplement: Supplementary file 1 — Additional file 1:Table S1. The Baseline Characteristics of participants on both sides of the inflection point. Table S2. The baseline characteristics of participants according to the clinical cut-off point for hemoglobin. Table S3. Relationship between Hb group and the renal composite endpoint in different models. [file 12882_2022_2920_MOESM1_ESM.docx]

**Association between hemoglobin and chronic kidney disease progression: a secondary analysis of a prospective cohort study in Japanese patients**

**Running title:** The relationship between hemoglobin and CKD progression

**Wushan Pan^1^**^#^**, Yong Han^2,3,^**^#^**, Haofei Hu^4,5^*,Yongcheng He^6^***

^1^Department of Nephrology, Kaifeng Central Hospital, Kaifeng 475000, Henan Province, China

^2^Department of Emergency, Shenzhen Second People’s Hospital, Shenzhen 518000, Guangdong Province, China

^3^Department of Emergency, The First Affiliated Hospital of Shenzhen University, Shenzhen 518000, Guangdong Province, China

^4^Department of Nephrology, Shenzhen Second People’s Hospital, Shenzhen 518000, Guangdong Province, China

^5^Department of Nephrology, The First Affiliated Hospital of Shenzhen University, Shenzhen 518000, Guangdong Province, China

^6^Department of Nephrology, Shenzhen Hengsheng Hospital, Shenzhen 518000, Guangdong Province, China

**^#^** Wushan Pan and Yong Han have contributed equally to this work.

*Corresponding author

Haofei Hu,

Department of Nephrology,

Shenzhen Second People’s Hospital,

No.3002 Sungang Road, Futian District,

Shenzhen 518000,

Guangdong Province,

China

Tel:+86-755-83366388

E-mail: huhaofei0319@126.com

Yongcheng He,

Department of Nephrology,

Shenzhen Hengsheng Hospital,

No. 20 Yintian Road, Baoan District,

Shenzhen 518000,

Guangdong Province,

China

Tel:+86-755-83366388

Fax:+86-755-83356952

E-mail: heyongcheng640815@126.com

## TableS1 The Baseline Characteristics of participants on both sides of the inflection point.

| Hb(g/dL) | <8.6 | >=8.6 | P-value |
| --- | --- | --- | --- |
| Participants | 53 | 909 |  |
| Age(years) | 72.85 ± 10.79 | 67.03 ± 13.64 | 0.002 |
| SBP(mmHg) | 141.69 ± 21.15 | 139.25 ± 21.90 | 0.430 |
| BMI(kg/m^2^) | 22.83 ± 5.16 | 23.81 ± 4.00 | 0.090 |
| Hb(g/dL) | 7.78 ± 0.64 | 12.30 ± 2.01 | <0.001 |
| ALB(g/dL) | 3.35 ± 0.63 | 3.90 ± 0.59 | <0.001 |
| Scr(mg/dL) | 2.80 (2.17-4.25) | 1.65 (1.20-2.49) | <0.001 |
| eGFR (ml/min per 1.73 m2) | 20.41 ± 15.80 | 33.77 ± 17.86 | <0.001 |
| UPCR (g/gCr) | 1.79 (0.70-4.33) | 0.64 (0.11-2.60) | 0.025 |
| Gender |  |  | 0.034 |
| Male | 30 (56.60%) | 640 (70.41%) |  |
| Female | 23 (43.40%) | 269 (29.59%) |  |
| Etiology of CKD |  |  | 0.188 |
| Diabetic nephropathy, n(%) | 20 (37.74%) | 224 (24.64%) |  |
| Nephrosclerosis, n (%) | 18 (33.96%) | 367 (40.37%) |  |
| Glomerulonephritis, n (%) | 7 (13.21%) | 171 (18.81%) |  |
| Other, n (%) | 8 (15.09%) | 147 (16.17%) |  |
| Urinary occult blood, n(%) | 15 (28.30%) | 295 (32.45%) | 0.530 |
| Hypertension, n (%) | 49 (92.45%) | 817 (89.88%) | 0.543 |
| History of CVD, n (%) | 20 (37.74%) | 238 (26.18%) | 0.065 |
| Diabetes, n (%) | 27 (50.94%) | 337 (37.07%) | 0.043 |
| Use of RAAS inhibitor, n(%) | 39 (73.58%) | 587 (64.58%) | 0.181 |
| Use of calcium channel blocker, n (%) | 31 (58.49%) | 428 (47.08%) | 0.106 |
| Use of diuretics, n (%) | 25 (47.17%) | 287 (31.57%) | 0.018 |

Continuous variables are presented as mean ± standard deviation and median with interquartile ranges. Categorical data are presented as numbers and percentages.

Abbreviations: BMI, body mass index; SBP, Systolic blood pressure; Scr, Serum creatinine; ALB, Serum albumin; HB, Hemoglobin; CKD, chronic kidney disease; CVD, cardiovascular disease; eGFR, estimated glomerular filtration rate; UPCR, urinary protein/creatinine ratio; g/gCr, gram per gram creatinine; RAAS, renin-angiotensin aldosterone system.

## Table S2. The baseline characteristics of participants according to the clinical cut-off point for hemoglobin

| HB(g/dL) | <10 | >=10, <12 | >=12 | P-value |
| --- | --- | --- | --- | --- |
| Participants | 173 | 290 | 499 |  |
| Age(years) | 71.64 ± 12.08 | 69.46 ± 13.11 | 64.64 ± 13.72 | <0.001 |
| SBP(mmHg) | 141.04 ± 21.83 | 140.14 ± 22.13 | 138.38 ± 21.69 | 0.303 |
| BMI(kg/m^2^) | 23.11 ± 4.65 | 23.68 ± 4.01 | 24.03 ± 3.87 | 0.035 |
| Hb(g/dL) | 8.87 ± 0.87 | 10.94 ± 0.57 | 13.81 ± 1.30 | <0.001 |
| ALB(g/dL) | 3.50 ± 0.64 | 3.74 ± 0.54 | 4.08 ± 0.54 | <0.001 |
| Scr(mg/dL) | 2.66 (2.07-4.17) | 2.12 (1.54-3.11) | 1.30 (1.07-1.73) | <0.001 |
| eGFR (ml/min per 1.73 m2) | 19.81 ± 13.21 | 24.72 ± 13.26 | 42.46 ± 16.54 | <0.001 |
| UPCR (g/gCr) | 1.65 (0.55-4.22) | 1.38 (0.22-3.58) | 0.28 (0.06-1.25) | <0.001 |
| Gender |  |  |  | <0.001 |
| Male | 96 (55.49%) | 178 (61.38%) | 396 (79.36%) |  |
| Female | 77 (44.51%) | 112 (38.62%) | 103 (20.64%) |  |
| Etiology of CKD |  |  |  | <0.001 |
| Diabetic nephropathy, n(%) | 69 (39.88%) | 98 (33.79%) | 77 (15.43%) |  |
| Nephrosclerosis, n (%) | 61 (35.26%) | 102 (35.17%) | 222 (44.49%) |  |
| Glomerulonephritis, n (%) | 21 (12.14%) | 49 (16.90%) | 108 (21.64%) |  |
| Other, n (%) | 22 (12.72%) | 41 (14.14%) | 92 (18.44%) |  |
| Urinary occult blood, n(%) | 51 (29.48%) | 121 (41.72%) | 138 (27.66%) | <0.001 |
| Hypertension, n (%) | 163 (94.22%) | 274 (94.48%) | 429 (85.97%) | <0.001 |
| History of CVD, n (%) | 66 (38.15%) | 87 (30.00%) | 105 (21.04%) | <0.001 |
| Diabetes, n (%) | 86 (49.71%) | 123 (42.41%) | 155 (31.06%) | <0.001 |
| Use of RAAS inhibitor, n(%) | 118 (68.21%) | 213 (73.45%) | 295 (59.12%) | <0.001 |
| Use of calcium channel blocker, n (%) | 95 (54.91%) | 153 (52.76%) | 211 (42.28%) | 0.002 |
| Use of diuretics, n (%) | 87 (50.29%) | 117 (40.34%) | 108 (21.64%) | <0.001 |

Continuous variables are presented as mean ± standard deviation and median with interquartile ranges. Categorical data are presented as numbers and percentages.

Abbreviations: BMI, body mass index; SBP, Systolic blood pressure; Scr, Serum creatinine; ALB, Serum albumin; HB, Hemoglobin; CKD, chronic kidney disease; CVD, cardiovascular disease; eGFR, estimated glomerular filtration rate; UPCR, urinary protein/creatinine ratio; g/gCr, gram per gram creatinine; RAAS, renin-angiotensin aldosterone system.

**Table S3. Relationship between Hb group and the renal composite endpoint in different models**

| Exposure | Crude model (HR,95%CI, P) | Model I(HR,95%CI, P) | Model II (HR,95%CI, P) |
| --- | --- | --- | --- |
| HB group |  |  |  |
| 10-12g/dL | 1.0 | 1.0 | 1.0 |
| ≥12g/dL | 0.259 (0.189, 0.356) <0.00001 | 0.250 (0.180, 0.347) <0.00001 | 0.602 (0.425, 0.853) 0.00434 |
| <10g/dL | 1.612 (1.212, 2.144) 0.00102 | 1.584 (1.181, 2.124) 0.00214 | 1.230 (0.896, 1.688) 0.19989 |

Crude model: we did not adjust other covariants

Model I: we adjust age, gender, BMI, SBP, hypertension, diabetes, history of CVD

Model II: we adjust age, gender, BMI, SBP, hypertension, diabetes, history of CVD, UPCR, eGFR, ALB, urinary occult blood, use of RAAS inhibitor, use of calcium channel blocker, use of diuretics

CI:confidence, Ref:reference
